# Personalization strategies in digital mental health interventions: a systematic review and conceptual framework for depressive symptoms

**DOI:** 10.3389/fdgth.2023.1170002

**Published:** 2023-05-22

**Authors:** Silvan Hornstein, Kirsten Zantvoort, Ulrike Lueken, Burkhardt Funk, Kevin Hilbert

**Affiliations:** ^1^ Department of Psychology, Humboldt-Universität zu Berlin, Berlin, Germany; ^2^ Institute of Information Systems, Leuphana University, Lueneburg, Germany

**Keywords:** depression, digital mental health, personalization, precision care, iCBT, machine learning

## Abstract

**Introduction:**

Personalization is a much-discussed approach to improve adherence and outcomes for Digital Mental Health interventions (DMHIs). Yet, major questions remain open, such as (1) what personalization is, (2) how prevalent it is in practice, and (3) what benefits it truly has.

**Methods:**

We address this gap by performing a systematic literature review identifying all empirical studies on DMHIs targeting depressive symptoms in adults from 2015 to September 2022. The search in Pubmed, SCOPUS and Psycinfo led to the inclusion of 138 articles, describing 94 distinct DMHIs provided to an overall sample of approximately 24,300 individuals.

**Results:**

Our investigation results in the conceptualization of personalization as purposefully designed variation between individuals in an intervention's therapeutic elements or its structure. We propose to further differentiate personalization by what is personalized (i.e., intervention content, content order, level of guidance or communication) and the underlying mechanism [i.e., user choice, provider choice, decision rules, and machine-learning (ML) based approaches]. Applying this concept, we identified personalization in 66% of the interventions for depressive symptoms, with personalized intervention content (32% of interventions) and communication with the user (30%) being particularly popular. Personalization via decision rules (48%) and user choice (36%) were the most used mechanisms, while the utilization of ML was rare (3%). Two-thirds of personalized interventions only tailored one dimension of the intervention.

**Discussion:**

We conclude that future interventions could provide even more personalized experiences and especially benefit from using ML models. Finally, empirical evidence for personalization was scarce and inconclusive, making further evidence for the benefits of personalization highly needed.

**Systematic Review Registration:**

Identifier: CRD42022357408.

## 1. Introduction

At an estimated lifetime prevalence of more than 10% (1, 2), major depressive disorder (MDD) is the second leading cause of years lived in disability (3). While this makes efficient treatments urgently needed, traditional approaches such as face-to-face psychotherapy are difficult to access for a significant part of patients (4–6). However, providing treatment through digital channels such as mobile applications and online formats (7) is effective in reducing depressive symptoms (8, 9) in a cost-effective way (10). Since most of the world population has access to the internet (11) and/or a smartphone (12), digital mental health interventions (DMHIs) bypass barriers to traditional treatment.

Despite their potential, DMHIs inherit some of the general problems in depression treatment: Estimates for treatment dropout, as observed in RCTs, are up to 50% when considering publication bias (13). Moreover, response rates are unsatisfactory at less than 50% (14). Therefore, improving outcomes and reducing dropouts in DMHIs are expected to be highly impactful in facing the burden of depression.

Luckily, DMHIs' unique delivery channel provides new opportunities to improve the treatment of those suffering from depressive symptoms. Specifically, digital applications can efficiently be individualized to improve users' experience and outcomes, as observable across many other domains, ranging from e-commerce (15) over e-learning (16) towards social media (17). Simultaneously, the importance of accommodating patients’ preferences for treatment outcomes in mental healthcare has been well established (18). Hence, the personalization of interventions to adapt treatment to individual needs is a promising approach to improving care, for depressive symptoms and beyond (19–22).

In line with that idea, a meta-analysis from 2013 showed that algorithm-based tailoring of DMHIs is associated with better outcomes (23). A review from 2022 found that none of the 26 reviewed apps for depression used just-in-time (JIT) adaptations, a mechanism for personalizing the timing of content delivery based on the individual or the situation (24). Another current systematic review investigated tailored interventions for workplace mental health (25), finding benefits on several outcomes when content or feedback was tailored towards the individual. Finally, a component network analysis examined the benefits of common internet-based cognitive behavioral therapy (iCBT) packages for depression, discovering small interactions between treatment components and patient characteristics (26).

While these publications are unified in their call for more personalization in DMHIs, they do not add up to a satisfactory empirical and theoretical ground for it. Firstly, the fragmented use of vocabulary fails to demarcate personalization from other distinct phenomena related to variability in DMHIs. For example, the term “tailoring” is used across various scopes and foci (23, 25, 27), while similar mechanisms are elsewhere called “individualized” (28) or “personalized” (26, 29). This diversity in vocabulary is shared with non-digital settings, as for traditional psychotherapy, 15 different terms for the same phenomena of varying treatment between individuals were reported (30). Secondly, in contrast to the breadth of used vocabulary, the focus of mechanisms within studies seems to be relatively narrow, focusing on specific mechanisms (23, 24) or areas (25, 28) of personalization. This potentially leads to an underestimation of variability already in place. Finally, while two of the mentioned reviews investigated the benefits of personalization through direct comparisons, they did so without a specific focus on depression and, related to the aforementioned narrow conceptualizations of personalization, with few studies being included. In conclusion, the concept, prevalence, and efficacy of personalization in DMHIs for depressive symptoms are not adequately delineated. Therefore, a disorder-specific review developing a conceptual framework for personalization and reviewing a wide span of interventions seems needed.

This systematic review aims to reduce the gap between the potential of personalization and its actual implementation by performing a comprehensive review of DMHIs for depressive symptoms with the following purposes:

1. Extract a conceptual framework that allows a clear and meaningful way of investigating, discussing, and classifying personalization.
2. Apply this framework to the available literature and report current use and mechanisms.
3. Evaluate the available evidence by identifying studies that directly compare interventions with different degrees of personalization.

## 2. Methods

This review was planned and reported following the Preferred Reporting Items for Systematic Reviews and Meta-Analyses (PRISMA) guidelines (31). The protocol of this review was registered in the International Prospective Register of Systematic Reviews of the National Institute for Health Research (PROSPERO) under the ID CRD42022357408. The protocol was updated once after initial piloting to improve the alignment of the inclusion criteria and data extraction method with the scope of the review. Specifically, a new classification dimension for personalization was added that occurred in the literature and did not fit the pre-defined schema and the exclusion of e.g., prenatal depression was added to improve the comparability between included interventions. The final version of the protocol can be found in the Supplementary Appendix S1.

### 2.1. Search strategy

In the first step, a search was performed in three major databases (SCOPUS, PubMed, PsycInfo) to identify all published studies on DMHIs for depressive symptoms. The full search strings can be found in Supplementary Appendix S2. Additionally, three related reviews (13, 14, 32) were screened, and studies not yet included were added. Finally, papers brought to the author's awareness by being discussed in our included articles, not included yet but fulfilling our selection criteria, were added.

### 2.2. Selection criteria

We included empirical studies on DMHIs specifically targeting depressive symptoms, determining the interventions target by authors' self-report. This covered both, patients with diagnosed major depressive disorder (MDD), as well as with subclinical levels of symptoms. To be considered a DMHI, interventions needed to be delivered through the internet and/or a smartphone. We included only empirical, peer-reviewed, English studies and conference articles with original data and patient cohort. To ensure a focus on the most relevant interventions for current use, we start our search from 2015 onwards.

To narrow down the focus of this review, studies on interventions targeting comorbid disorders such as anxiety were excluded. The same applied to those targeting a specific subtype of depression (e.g., prenatal depression), a single sub-symptom (e.g., rumination), or adolescent or elderly people (below 18 years or >64 years). Finally, those studies using digital technologies exclusively as a means of communication, such as one-on-one psychotherapy delivered via the web, were excluded as well.

### 2.3. Selection procedure

One of the researchers (S.H.) performed an initial screening based on the title and abstract of the studies identified through the search strategy. A second researcher (K.Z.) conducted the same procedure for a randomly chosen subset of 100 studies, resulting in excellent interrater reliability (0.94). The full description of the intervention was then read by both reviewers for all remaining papers to determine the final selection, extract interventions and code the variables of interest. Disagreements on any aspect of this process were solved by discussion between the reviewers until a consensus was reached. If full texts were unavailable, they were requested from the corresponding author. This occurred 12 times, with 8 of the articles made available on request.

### 2.4. Development of the conceptual framework

During the initial screening and before the update of the PROSPERO registration, we developed the proposed framework in an iterative process, considering usability, conceptual literature, and the observed interventions. Specifically, we discussed how we could classify personalization in a way that allows us to not just cover all mechanisms in the literature but also maximize usability by defining the dimensions as distinct as possible. We did this as we needed a method to classify personalization mechanisms during the systematic review and we could not find a satisfactory framework in the literature yet.

We departed from a common dictionary definition defining personalization as “the action of designing or producing something that meets someone's individual requirement” (33). Based on that, we intended to classify personalization in DMHIs in a broad enough way to cover the diversity of mechanisms present in related reviews and studies. At the same time, we intended to narrow down the concept to those mechanisms affecting the therapeutic content and structure, setting it apart from superficial sources of variability. Therefore, we excluded interactivity (34), the sole replay of user input as part of the app experience. For example, showing each patient their previously set goal might be a powerful tool, but it does not change the underlying therapeutic elements delivered. Additionally, we factored out customization (35), minor aesthetic adaptation such as users ability to change the color of an avatar. Finally, seeing personalization as referring to the level of the individual patient, we excluded group-based variability, such as cultural adaptation of the entire intervention (36).

Numerous screened interventions used a structured session-based approach to deliver their intervention—a common approach among manualized mental health interventions (37). Therefore, we identified (a) content (what is delivered during a session) and (b) order (how sessions are ordered) as potential areas of personalization. Since (c) guidance (level of human contact) is a highly relevant and variable aspect of DMHIs (38) we added it as another dimension. Finally, as we discovered prompts and mechanisms targeting the timing of interventions not being sufficiently represented in these three categories, we appended (d) communication as another dimension.

While, as mentioned above, we intended to exclude customization as minor user-choice-based adaptations of the intervention, we did not exclude user choice *per se* from our concept. This differs from the use in fields like marketing, where anything done by the user is defined as customization, not personalization (35). However, we saw the inclusion of actively designed user choice being justified for the following reasons: Firstly, user choice was a common mechanism described in the included interventions. Secondly, those mechanisms seem easily implementable and therefore highly relevant for practitioners interested in personalizing their intervention. Finally, user agency has been shown to be particularly relevant in mental healthcare (18). We also identified provider choice as another mechanism for guided and blended interventions. For data-driven personalization mechanisms, we saw rule-based and ML as distinct mechanisms applying static or learning criteria for personalization.

### 2.5. Data extraction

The framework developed above was applied to all identified interventions, coding the presence of personalization for each of the four (a–d) dimensions and classifying the underlying mechanism. For this, interventions had to be extracted from the included studies, and information from several studies on the same intervention had to be merged. If more than one distinct intervention was presented in a study, they were coded separately. Intervention versions in different languages were not coded separately if not reported to be clearly distinct in their content. If more than one study was available, a single observation of personalization resulted in a positive coding, but conflicting information was noted. Additionally, cited material such as older papers, weblinks, or appendices were consulted in the refrained from additional free-hand research on the reported interventions. In case information was indicative of personalization but insufficient for our coding, we contacted the corresponding author and asked for clarification. For this, we provided a four-week response window, including one reminder. Out of the seven authors contacted, six responded by providing additional information. In the single case where authors did not respond (39) we decided to code restrictively and assume the simpler of the potential mechanisms involved (rule-based instead of ML). Finally, for evaluating the evidence for personalization, we included every study that directly compared intervention versions that differed in their degree of personalization, according to our framework. We extracted effect sizes, dependent variables, and sample sizes for those.

## 3. Results

### 3.1. Study selection

**Figure 1:**
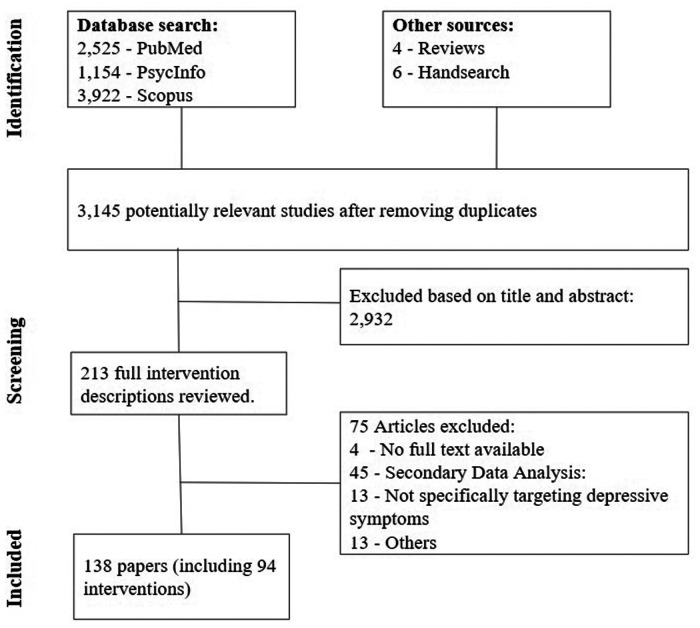
PRISMA flowchart of study inclusion.

Overall, we identified 3.143 potentially relevant publications and screened the title and abstract. For 213 of those, the full intervention description was reviewed, resulting in the final inclusion of *N* = 138 papers describing *k* = 94 distinct DMHIs for depressive symptoms (see Figure 1) (39–175).

### 3.2. Intervention and study characteristics

While mostly one study per intervention was included, for some up to seven publications on distinct trials were present and kept for further analysis. Across all studies, the reviewed interventions were deployed to approximately 24.300 participants, with an average sample size of 259 participants per intervention (range 1–2964). 75 of the interventions were used in a randomized controlled trial, with the remaining evidence coming from feasibility studies, naturalistic routine care data, and other study designs.

Most interventions had a duration between 6 and 12 weeks, and around 40 of the interventions report a structured module-/ session-based design, delivering the content in pre-defined blocks. Finally, 38 interventions were unguided (no human contact within the intervention), 32 guided (including guidance from clinician or coach), and 14 blended (combining face-to-face and digital treatment), with the remaining 10 covering more than one of those categories. An overview of all characteristics can be found in Supplementary Appendix S3.

### 3.3. Conceptual framework

**Figure 2:**
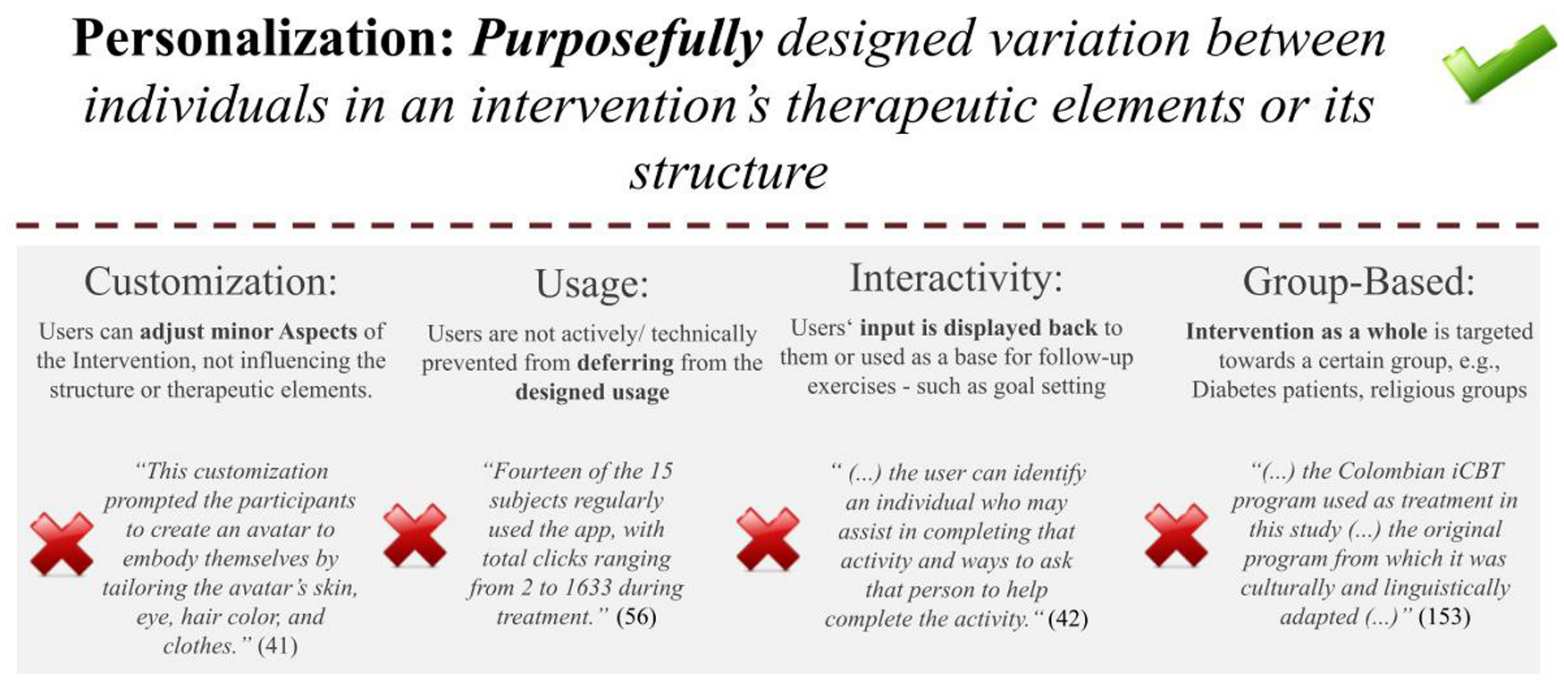
Personalization in comparison to the terms usage, customization, interactivity and group-based adaption.

A conceptual framework of personalization in DMHIs was synthesized from the reviewed DMHIs and theoretical considerations. In summary, an understanding of personalization as *purposefully designed variation between individuals in an intervention's therapeutic elements or its structure* emerged. As such, personalization is differentiated from customization, usage, interactivity, and group-based adaptations. Customization describes minor adjustments, such as visual aspects, leaving the actual therapeutic ingredients unchanged. Usage refers to possible user-induced differences in app usage that were not actively or purposefully designed. For example, variability in the time spent on a module is usage, the offering of short and long versions of a module qualifies as personalization. Interactivity, the mere replay of user input as for example commonly used for goal-setting exercises, as this leaves the actual therapeutic elements and structure unchanged. Finally, as we understand personalization as referring to the level of the individual, we see it as being distinct from group-based variability, such as the adaptation for a particular cultural context (see Figure 2).

**Figure 3:**
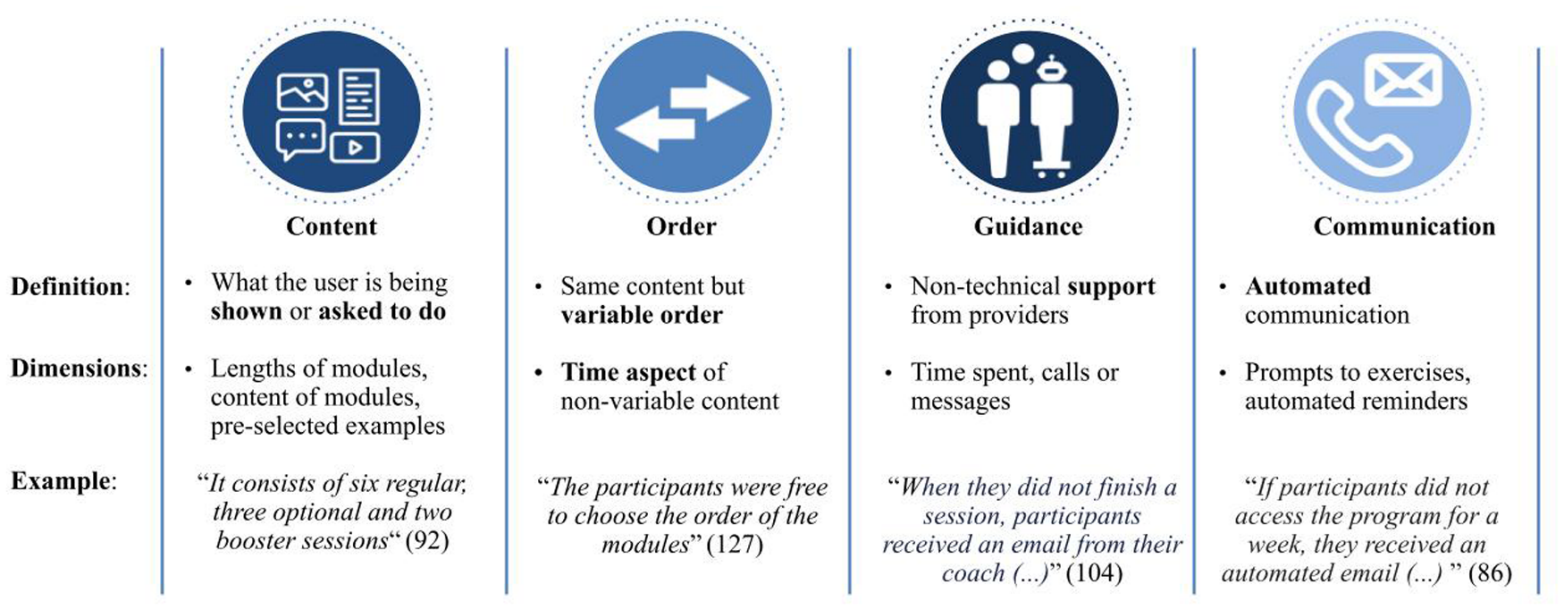
Dimensions of personalization.

Within our definition of personalization, four personalizable intervention dimensions emerged, namely content, guidance level, order, and communication, as summarized in Figure 3. Content describes all variability in the delivered intervention material, such as exercises, psychoeducative material or topics presented. Order includes cases when patients receive the same content but in different order. Guidance refers to the extent of therapeutic support offered. Communication concerns the channel, timing, and content of actively offered information outside of the intervention's content. This primarily includes prompts or reminder messages. Mechanisms regarding the frequency and timing of the intervention, such as JIT mechanisms, also fall under communication.

**Figure 4:**
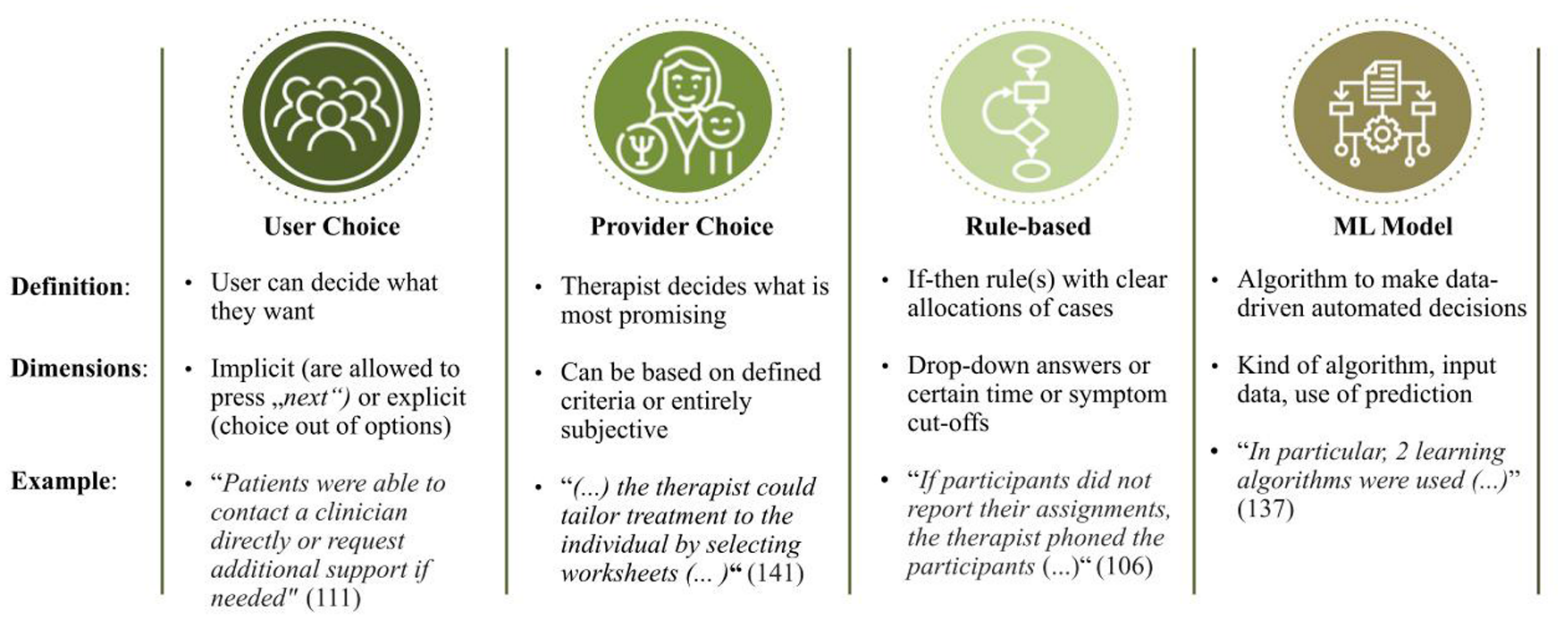
Mechanisms of personalization.

Further, four different mechanisms beyond personalization emerged: user choice, provider choice, rule-based and ML-based personalization (see Figure 4). User choice covers intentionally designed personalization based on the direct choice of the participant. For provider choice, either the individual providing guidance, or the clinician involved in a blended setting makes the personalization decision. Among automated personalization mechanisms, rule-based (if-then-decision rules) from Machine Learning (decisions with “learned” decision criteria) personalization mechanisms are gathered.

### 3.4. Results on personalization

Applying the proposed framework for classifying variability in DMHIs, personalization was reported for 62 of the 94 interventions (66%). Most prominently, personalization mechanisms were used in the content for 30 of the interventions (32%). This was followed by personalized communication (30%), type (25%), and order (4%). 43 of the 62 (69%) interventions with a reported personalization mechanism did so for only a single dimension, while one DMHI reported a mechanism for all four subdomains of their intervention (60–66).

**Figure 5:**
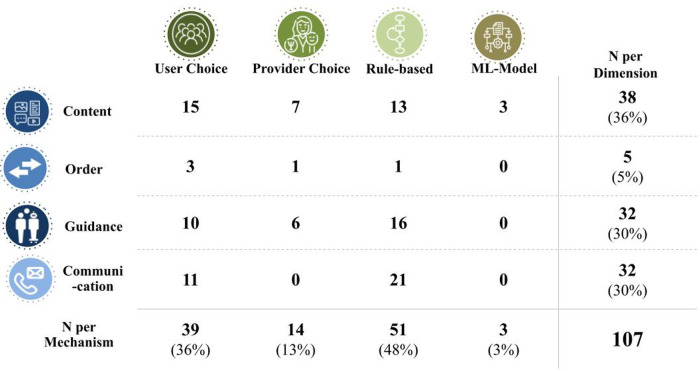
Personalization mechanisms per dimension of the intervention.

Across the 107 reported personalization mechanisms, rule-based was most prominent, being used in 51 cases (48%). User choice was observed in 39 cases (36%), and providers were involved in personalization 14 times (13%). The use of machine learning was reported three times (3%). Rule-based personalization was particularly prominent in the communication domain, accounting for 21 occurrences. Similarly, human guidance was personalized using decision rules 16 times. For content, user choice had a more prominent role, being reported 15 times. The use of personalization is summarized in Figure 5, with examples of the 3 most strategies being presented in Table 1. The share of interventions applying at least one personalization mechanism was the highest for guided interventions (72%), followed by unguided (63%) and tailed by blended (57%) interventions. Generally, the dimensions of personalization were equally spread across guidance levels. However, provider choice was nearly twice as common for blended than for guided interventions.

**Table 1:** Most prominent personalization strategies.

Number of interventions	Dimension	Mechanism	Description
21	Communication	Rule-based	e.g., Reminder for inactivity/non-completion
16	Guidance	Rule-based	e.g., Increased guidance/clinician contact for symptom changes.
15	Context	User choice	e.g., Optional content selectable for patient.

### 3.5. Use of automated decisions for personalization

Among the 55 automated mechanisms used, most were rule-based mechanisms of personalization. Here, activity data was heavily utilized, for example, for reminders in case of inactivity. Another common pattern was the use of symptom scores like the PHQ to step up care in the form of additional guidance (57) or the change from guided to blended care (169). While those approaches mostly used overall symptom severity, one exemption was the personalization based on suicide risk as e.g., in the form of additional prompts (146).

We identified three clear use cases of ML techniques for personalization. Firstly, EmoRecorder (70) used an activity recommendation system based on diverse data sources like app activity, sensor data and past recommendations. However, the intervention was at an early stage, being tested on a sample of only 15 healthy individuals. Secondly, the intervention MOSS (136) built on a JIT framework to assign intervention content depending on users' context and preferences. As such, it tested a recommender system with a sample of 126 adults. A third recommender system approach, so-called MUBS (137), applied a combination of ML and user choice by providing the 17 patients with a set of content recommendations.

### 3.6. Direct empirical comparison of more and less personalized interventions

Among the 138 papers in the final review, we identified two papers that included a direct comparison of a more and a less personalized version of an intervention. One study had participants fill out a questionnaire on motivational schemata and either matched them with an intervention arm to fit their motivational preference or a general one (40). Results showed effects for one of the two included motives (“being supported”) on anticipated adherence, working alliance, and satisfaction; however, the overall sample size of this trial was just 55 participants. Secondly, a study compared a program version including JIT prompts with one without those prompts, therefore, differing the personalization in the communication domain between trial arms (93). While both versions showed significant effects compared to the waitlist, no effects were reported between the arms. Again, this should be interpreted with caution, considering the sample size of around 60 individuals per group.

## 4. Discussion

In recent years, personalization has been widely discussed as a promising avenue to improve DMHI adherence and outcomes. Nevertheless, it remains unclear what it entails and how it is used. In this review, we address this need for the case of depressive symptoms, by defining personalization as purposefully designed variation in intervention content, order, guidance, or communication. As possible mechanisms to operationalize personalization, we extract user choice, provider choice, decision rules, and ML. Applying this framework to 94 interventions for depressive symptoms reveals that two-thirds use at least one technique for personalization. Especially rule-based personalization of communication and guidance and user choice-based personalization of content is common. However, among interventions applying personalization, a majority does so just for one out of four dimensions of the intervention. Also, the use of ML models is scarce and limited to feasibility studies. Additionally, just two of the included studies investigated the benefits of personalization, both having small samples and just one finding supporting evidence.

Arguably, the biggest contrast between the proposed potentials in the personalization of DMHIs (19–22) and the existing literature is the lack of implemented ML mechanisms. Several of the implemented non-learning algorithms and decision rules were well designed. Yet, literature on ML in DMHIs reveals ample further promising and feasible use cases. Firstly, a notable body of research provides encouraging results in outcome (176, 177) and dropout (178, 179) predictions in DMHIs. Adapting the interventions for assumed non-responders is a low-hanging fruit and has already been successful for other disorders (180). Secondly, a prominent algorithmic approach to personalization in digital products is recommender systems (181–183). While all included ML approaches were such recommender systems, they were in early stages and deployed to very small sample sizes. Finally, all included ML approaches focused on the content of the intervention. However, ML also is a promising approach to personalize guidance, communication and order.

Contrasting theory and observations in another dimension, the data used for personalization just samples a fraction of the technically possible. While app usage patterns are an obvious data option, smartphones can also measure sleep patterns (184), physical activity (185), social interactions (186), and many other data points known to be relevant for depressive symptoms. Readily available toolkits like Apple's health kit (187) reduce the effort for implementation significantly. However, particularly passive sensing was rarely utilized in the reviewed interventions. Notably, the potential of ML-based personalization is heavily intertwined with the quality of the data available to them. Beyond that, aspects such as ethical responsibility in health care and privacy rights must be strongly considered, especially when investigating automated decisions (188).

Several interventions used self-reported symptoms for the personalization of the intervention. Noticeably, these mechanisms mostly used overall symptom severity. This approach disregards that symptom profiles can vary massively between patients with the same overall score (189). Some evidence points toward distinct symptom patterns being associated with different optimal treatment procedures (190). Therefore, while overall severity seems reasonable for varying guidance or communication, the sub-symptoms might be a promising ground for personalizing content and order.

The two included trials that manipulated personalization did so with small sample sizes and inconclusive results. Subsequently, one barrier to implementing personalization might be the lack of clear evidence for its benefits. However, RCTs investigating personalization are likely costly and require large sample sizes when assuming smaller effect sizes than for waitlist-controlled studies. Luckily, meta-analytic approaches allow summarizing evidence across studies, even when personalization is rarely directly manipulated. While we mentioned one such approach investigating interactions between individuals and benefits of iCBT packages (26), we consider similar approaches for other personalization mechanisms as very promising. However the identification and comparison of relevant studies in meta-analyses requires shared vocabulary and a common framework. We believe that such future work will benefit from the shared conceptual framework proposed in this article.

There are some limitations of this review that should be considered. Firstly, published studies are just one marker of what interventions are in use. While several included interventions originated in a commercial setting, those from academic settings will likely still be overrepresented in this review. Secondly, we focused on personalization within an intervention, excluding the personalization of interventions themselves. For example, past approaches investigated the data-driven personalization of therapy school (191) or the decision between medication and CBT (192). Thirdly, identifying interventions for depressive symptoms while excluding those addressing comorbid disorders, particularly anxiety, has proven challenging. One example is when anxiety was mentioned as intervention target in a cited study, but not in the original paper. While this seems understandable in light of the well-established comorbidity of depression and anxiety (193), this resulted in several edge cases of inclusion. Fourthly, we took interactivity, customization, and group-based adaptions out of the scope of this review due to their difference in nature to personalization. This should not be misunderstood as an assumed inferiority, and we call for the further investigation of these approaches to complement or even substitute personalization. Fifthly, we did not evaluate our framework by any methods besides the literature review. Approaches like expert interviews could help to determine and improve the usability of the proposed conceptualization. Sixthly, to provide a wide and less biased picture of the state of personalization, a broad search strategy was used. However, studies using more specific terminologies might be underrepresented. For example, a study on ecological momentary interventions (EMI) was not identified by our search strategy (194) as EMI was not used as a search term. Also, as pointed out by one of the reviewers, the mesh term “Telemedicine” was not used. Future approaches could therefore benefit from the application of additional techniques for iterating on the search strategy, such as the wider use of sentinel articles. Finally, as we developed our framework exclusively with studies on depressive symptoms, it remains unclear whether there are more aspects to consider with other disorders. However, we expect this framework to provide value beyond the use case of depressive symptoms and encourage future studies to investigate personalization strategies in other domains.

In conclusion, our conceptual development and empirical evaluation holistically characterizes the current use of personalization for DMHIs for depressive symptoms. A broad conceptualization of personalization reveals that most interventions incorporate personalization mechanisms. However, we conclude that we are barely scratching the surface of what is technically possible and already gold standard in other research and business areas. At the same time, we see the thin empirical ground as a barrier to implementation and call for more direct and meta-analytic evidence to delineate the benefits personalization has over an “one size fits all”-approach. Finally, as we see this question as equally pressing for other disorders, we hope for similar-minded approaches for those in the future.

## Data Availability

A file with all included studies as well as the related coding regarding the variables of interest can be found in Appendix 3. A file with the full search strings used can be found in Appendix 2. A file containing all studies screened during the selection process is available on request.
