# Supplementary material for: Personalization strategies in digital mental health interventions: a systematic review and conceptual framework for depressive symptoms
Source: Front Digit Health. 2023 May 22;5:1170002. doi: 10.3389/fdgth.2023.1170002 (PMC10239832; doi:10.3389/fdgth.2023.1170002)
Supplement: Supplementary file 1 [file Datasheet1.pdf]

Supplementary Material

Personalization Strategies in Digital Mental Health Interventions: A Systematic Review and Conceptual Framework for Depressive Symptoms

Silvan Hornstein*, Kirsten Zantvoort, Ulrike Lueken, Burkhardt Funk, Kevin Hilbert

*** Correspondence:** Silvan Hornstein - silvan.hornstein@hu-berlin.de

1 Appendix 1: PROSPERO Protocol

1. Review Title:

Personalization Strategies in Digital Mental Health Interventions for Depression: A Systematic Review

2. Anticipated Start Date:

01.09.2022

3. Anticipated Completion Date:

31.03.2023

4. Stage of Review at time of this submission:

Not started yet.

5. Named Contact:

Silvan Hornstein

6. Named Contact Email:

silvan.hornstein@hu-berlin.de

7. Named Contact Address:

Faculty of Life Sciences, Department of Psychology, Humboldt-Universität zu Berlin, Berlin, Germany

8. Telefon

9. Organisational affiliation of the review

Humboldt-Universität zu Berlin, Arbeitsbereich Psychotherapie

10. Review team members and their organisational affiliations.

Prof. Dr. Ulrike Lueken, Humboldt-Universität zu Berlin, ulrike.lueken@hu-berlin.de

Dr. Kevin Hilbert, Humboldt-Universität zu Berlin, kevin.hilbert@hu-berlin.de

Kirsten Zantvoort, Leuphana Universität Lueneburg, kirsten.zantvoort@leuphana.de

Prof. Dr. Burkhardt Funk, Leuphana Universität Lueneburg, burkhardt.funk@leuphana.de

11. Funding sources/sponsors.

None

12. Conflict Of Interest

Silvan Hornstein is currently employed as Data Scientist by Elona Health, a start-up building blended mental healthcare solutions for the German market. Burkhardt Funk is a shareholder at HelloBetter, a digital mental health company developing digital interventions, and PersonalAIze, an AI consulting company.

13. Collaborators:

None

14. Review Question

Are Digital Mental Health Interventions for Depression static (= every patient is going through the same content in the same order) or adaptive/ dynamic (different content and order for patients)? If DMH Interventions are adaptive/ dynamic, how are content and ordering tailored to the individual patient?

15. Search Strategy

Databases: PubMed, PsycINFO and Scopus

Search will be performed between 01.09.2022 and 31.10.2022. Only publications published in English in peer-reviewed journals and conference proceedings will be included.

Additionally, reviews identified fulfilling the inclusion criterion defined below will be used as additional source to identify relevant literature. For this, the cited literature in these reviews will be scanned for missing papers not included in this review yet.

Search String for Pubmed:

(("Depression"[Mesh] OR "Depressive Disorder"[Mesh]) OR (depress*[ti])) AND ((smartphone*[ti]) OR (mobile[ti]) OR (phone[ti]) OR (app*[ti]) OR (web[ti]) OR (internet[ti]) OR (digital[ti]) OR (online[ti]) OR (web-based[ti]) OR (iCBT[ti]) OR (computer[ti]) OR (“digital technology”[Mesh]) OR (“mobile applications”[Mesh]) OR (“Smartphone”[Mesh]))

An equivalent search strategy will be used for the other databases.

16. Search Strategy Link

-

17. Condition being Studied.

Major Depressive Disorder (MDD)/Elevated Depressive Symptoms.

18. Participants/population

For this review, studies will be selected based on intention of the intervention and not the actual tested population. Therefore, an intervention designed for insomnia but tested with a population of depressed individuals would be excluded, while an intervention targeting depression being tested in e.g. a sample of university students will be included. The following markers will be used to determine the intended use of an intervention:

1. Language: A sentence like 'Intervention for depression' would indicate inclusion, 'Intervention for depression and anxiety' exclusion

2. Used questionnaires: The use of an anxiety questionnaire as the main outcome would indicate exclusion.

19. Intervention(s)/Exposure(s)

Digital Mental Health interventions are those that deliver care via digital channels such as smartphones or the internet. DMH interventions can be guided (including contact with a clinician), self-guided (no contact with a clinician) or blended (DMH intervention integrated with other forms of treatment, such as psychotherapy).

This review does focus on interventions intended for use in the direct treatment of symptoms, excluding those that are solely focussing on prevention, relapse prevention, post intervention care or passive monitoring of symptoms.

Additionally, interventions need to deliver content and/or exercises and not be solely based on digital human support.

Finally, this review focuses on interventions designed specifically for depression and depressive symptoms.

Therefore, interventions that target depression and anxiety, or any other comorbid disorder are excluded. Additionally, interventions targeting a specific sub-symptom of depression (e.g. rumination) are excluded. Finally, studies on interventions targeting a specific subtype of depression, such as prenatal depression are excluded.

20. Comparators:

None

21. Type of Studies:

1. Study addresses internet/smartphone based intervention

2. Intervention is specifically and exclusively targeting depressive symptoms and/or MDD, which is measured by an established diagnostic questionnaire (scores in established clinical questionnaires or interviews such as the PHQ or HAMA that indicate at least mild depressive symptoms, following standard cut off criteria) and/or MDD (self-assigned or diagnosed according to DSM-IV TR / DSM-5 /ICD-10 diagnostic criteria).

3. Intervention is not targeting solely a specific subsymptom of depression (e.g. rumination). Neither is the intervention targeting a specific subtype of depression, or designed for prevention, monitoring or post-intervention-care.

4. Empirical Study with original data.

5. Publication in a peer-reviewed journal

6. Published in 2015 or later

7. In English language

8. Intervention is designed for Adults aged between 18 and 70, and therefore not exclusively for adolescent or elderly people.

9. Full Text available

10. Protocols and Secondary Data Analyses are excluded.

22. Context:

As this review is interested in the interventions described in the articles selected, in a second step interventions need to be extracted from the selected articles. As different studies might investigate the same intervention or single studies compare more than one intervention, the extracted interventions will likely differ in form and number from the selected articles. The following criteria will be applied to extract interventions from the selected studies

If several articles use the same intervention

1. The newest article will be used to determine all datapoints outside of the main variables, such as duration of the intervention.

2. All articles will be used to determine the variable of interests. In case of opposing information, the newer article will be used, but the discrepancy mentioned in a comment.

3. If there is more than one intervention evaluated in one article, they will be included as separate interventions for additional analysis, as long as every intervention is clearly distinct from each other and fulfills the inclusion criteria

23. Main Outcome(s):

Is the intervention static or adaptive? This is determined by the description of the intervention in the methods section. Variation will be coded for the following subdomains.

1. Content

2. Order

3. Communication

3. Level of Guidance

For variable subdomains, it is coded what the source of variation is (e.g. patient or clinician) and what the mechanism of variability (e.g. choice, algorithm..).

For the coding all available papers on the intervention are used and inconsistency handled as described at 23.

In absence of information on a variable aspect in a subdomain, this domain is coded as being static. If variability/tailoring/personalisation is mentioned but not explained, authors of the study will be contacted and asked for clarification with a 4 week window to respond and one reminder after 2 weeks.

24. Additional Outcome(s)

It will also be investigated, whether there are any studies including direct comparisons of adaptive and non-adaptive interventions. It will be reported

1. How much of these studies exist

2. Whether the superiority of adaptive interventions was proven

3. The effect sizes.

25. Data Extraction (Selection and Coding)

Study Selection:

The titles and abstracts of the results of the database search will be checked and studies selected as described at 22.

Duplicates will be removed. For a random subsample of 100 studies, this will also be done by a second researcher, in order to calculate interrater reliability. In case of an insufficient interrater reliability (<0.9), the second reviewer will do the whole filtering process, and disagreement between the two researchers will be discussed and decided by consensus.

In the next step, distinct interventions will be extracted from the selected articles and data extracted as described at 23. and 24. This will be done by 2 reviewers independently and disagreement solved by consent.

The method section of all papers on an intervention is used to determine the main outcomes, as described at 23. Also, linked additional material will be included as well. No additional research will be performed on characteristics of the intervention, with exemption of contacting of authors as described at 24.

The following variables will be extracted:

Name of Intervention

Year (Newest)

Owner

Level of Guidance(Guided/Unguided/Blended)

Delivery

Content (Short Summary)

Real World Data (Yes/No)

Main Outcome: Staticness, Source of Variability, Mechanism of Variability

Secondary outcome: Effect measures in case of direct comparison of adaptive/non adaptive intervention.

26. Risk of bias (quality) assessment

The amount of authors responding to our clarification request will be reported.

27. Strategy for Data Synthesis

The following results will be reported:

1. Amount of studies being variable in % (overall as well as per subdomain).

2. Type of variability in %.

3. Source of variability in %.

4. Variability by type of intervention

5. Narrative summary of most common mechanisms of introducing variability.

28. Analysis of subgroups or subsets

Interventions will be subdivided by type of digital mental health intervention, i.e., self-guided, guided and blended. As this type influences the potential techniques to introduce variability into the program, this appears relevant to be coded and analyzed.

29. Type and Method of the Review.

Systematic Review. Mental health and behavioral conditions

30. Language

English

31. Country

Germany

32. Other registration details

None

33. Reference and/or URL to published protocol

-

34. Dissemination Plan

35. Keywords

Digital Mental Health, Depression, Precision Care, Precision Psychotherapy

36. Details of any existing review of the same topic by the same authors.

None

37. Current Review Status

Not started

38. Any Additional Information

39. Details of final reports/publications
