# Supplementary material for: Personalization strategies in digital mental health interventions: a systematic review and conceptual framework for depressive symptoms
Source: Front Digit Health. 2023 May 22;5:1170002. doi: 10.3389/fdgth.2023.1170002 (PMC10239832; doi:10.3389/fdgth.2023.1170002)
Supplement: Supplementary file 2 [file Datasheet2.pdf]

Meta Data										Content Variability		Order Variablity		Guidance Variability		Comunication Variability	
**Name**	**Papers**	**PaperCount**	**Year (newest)**	**Type**	**Duration in weeks**	**Study Type**	**Sample Size**	**Content**	**Direct Comparison**	**Content variable**	**Source of Variability**	**Order variable**	**Source of Variability**	**Type variable**	**Source of Variability**	**Comunication variable**	**Source of Variability**
Adapted version of mood	10.2196/37287	1	2022	Unguided	Other	Other	55	Other	Yes	1	rule-based	0		1	rule-based	0	
Adventure Self	10.1016/j.invent.2017.04.001	1	2017	Blended	10	RCT	23	Group Intervention	No	0		0		0		0	
AirHeart	10.2196/39516	1	2022	Unguided	2	RCT	94	Module-based.	No	0		0		0		1	user choice
Aptivate! / Moodivate	10.1016/j.jad.2019.03.009 10.1016/j.beth.2018.12.003	2	2019	Unguided	8	RCT/Feasibility	46	Behavioural Activation Intervention	No	0		0		0		0	
ASCENSO	10.2196/26814	1	2021	Blended	36	RCT	84	Other	No	0		0		1	rule-based, user choice	1	rule-based
Be Good to Yourself	10.1016/j.psychres.2018.08.113	1	2018	Unguided	4	RCT	45	40 Single Exercises	No	1	user choice	0		0		1	user choice
Beating the Blues	10.1016/j.beth.2017.04.004, 10.1176/appi.ps. 201900283, 10.1093/ofid/ofaa280, 10.2196/27630, 10.3310/hta191010	5	2020	Guided	8/12	RCT/ RCT/	485	Module-based.	No	1	user choice	0		1	user choice, provider choice	0	
BlueWatch	10.2196/mental.9445	1	2018	Unguided	6	Usability Testing	5	Module-based.	No	0		0		0		0	
Boost Me	10.1037/ser0000261	1	2019	Guided	6	RCT	10	Behavioural Activation Intervention	No	0		0		0		0	
CBM Intervention	10.1016/j.jad.2015.02.026, 10.1177 /2167702614560746, 10.1016/j.brat.2016.07.008	3	2016	Unguided	1/4	RCT	136	CBM	No	0		0		1	rule-based	0	
CBT MobileWork	10.1007/s10608-020-10159-4	1	2020	Blended	Other	Pre-Post	23	homework assg.	No	1	rule-based	0		1	rule-base	0	
CBT-M	10.2196/24380	1	2021	Guided	25	RCT	22	Module-based.	No	0		0		0		0	
Depressioncoach (forr	10.1159/000481515	1	2020	Guided	6	RCT (no control)	1089	Module-based	No	0		0		0		0	
Depressionshjälpen	10.1080/02813432.2017.1333299	1	2018	Guided	12	RCT	45	Module-based	No	0		0		1	user choice	0	
Deprexis	10.1037/ccp0000171, 10.1159/000481177, 10.1055/a-1826-2888, 10.1159/000445355, 10.1186/s13561-020-00273-0, 10.1016/j.invent. 2014.12.003, 10.1016/S2215-0366(14)00049-2	7	2022	Guided	12	RCT	2964	Module-based.	No	1	rule-based	1	rule-based	1	rule-based	1	rule-based
e-couch	10.2196/mental.4356	1	2016	Unguided	12	Pre-Post	298	Other	No	0		0		0		0	
EFMT	10.1038/s41746-018-0025-5, 10.1177 /2470547019877880	2	2019	Unguided	6	RCT	42	EFMT	No	1	rule-based	0		0		0	
EmoRecorder	10.4108/eai.14-10-2015.2261655	1	2015	Unguided	2	Other	15	RecommenderSys	No	1	ML model	0		0		0	
eSMART-MH	10.1111/ppc.12112	1	2015	Guided	12	RCT	30	VR	No	0		0		0		0	
GAF-ID	10.1016/S2215-0366(18)30223-2	1	2018	Guided	8	RCT	159	Module-based.	No	0		0		0		1	user choice
GET.ON Mood Enhanc	10.1159/000438673 10.1016/j.jad.2018.12.065 10.1016/j.beth.2017.05.004 10.1016/j.invent. 2021.100455	4	2021	Guided	6	RCT	550	Module-based.	No	1	user choice, provider choice	0		0		1	user choice, rule-based
GIAI	10.1097/MD.0000000000018774	1	2020	Guided	10	RCT	96	Unclear	No	1	provider choice	0		1	user choice	0	
Good Days Ahead	10.1176/appi.ajp.2017.17010089, 10.1001 /jamanetworkopen.2021.46716	2	2022	Guided	12/16	RCT	172	Module-based.	No	0		0		0		1	user choice
HeadGear	10.2196/11661	1	2018	Unguided	4	PrototypeTestin g/Feasibility	105	Single Tasks, BA & Meditation	No	0		0		0		0	
Headspace	10.1016/j.invent.2021.100413, 10.1177 /1046878119851821	2	2021	Guided/Unguide d	2	RCT, PrePost	89	Meditation	No	0		0		0		0	user choice
Healthy Psychological	10.1017/S0033291722000599	1	2022	Guided	5	RCT	110	module-based.	No	0		0		0		0	
Help4Mood	10.1177/1357633X15609793	1	2016	Blended	4	RCT	13	Other	No	1	rule-based,user choice	0		0		1	rule-based
HelpID	10.2196/jmir.6546	1	2017	Guided	12	RCT	90	Other	No	0		0		1	user choice	0	
Hermes	10.1016/j.brat.2022.104070	1	2022	Guided/Unguide d	8	RCT	316	Other	No	0		0		1	rule-based	0	
HLP	10.2196/15845	1	2020	Blended	4-8	RCT	54	Module-based	No	0		0		0		1	rule-based
IACT china	10.1002/jclp.23329	1	2021	Unguided	6	RCT	95	Module-based	No	0		0		0		1	rule-based
iFightDepression	10.1016/j.invent.2021.100476, 10.1177 /1357633X221084584, 10.1016/j.invent. 2022.100551,10.2196/28321	4	2022	Guided/Unguide d	6	Routine Care Data, RCT	2640	Module-based	No	0		0		0		0	
IMI eSano BackCare-I	10.1016/j.jad.2022.04.004	1	2022	Guided	9	RCT	105	Module-based	No	1	user choice	0		0		0	
ImproveyourMood plus	10.1016/j.jad.2021.05.021	1	2021	Unguided	3	RCT	122	JIT	Yes	0		0		0		1	rule-based
IP Stockholm	10.1371/journal.pone.0161191	1	2016	Guided	9	Routine Care Data	1738	Module-based	No	0		0		0		0	
iPST	10.1136/bmjinnov-2015-000098, 10.2196/10130	2	2018	Unguided	12	RCT	323	Other	No	1	rule-based	0		0		0	
Lifestyle Hub	10.1037/ccp0000695	1	2021	Unguided	8	RCT	39	Module-based	No	0		0		0		1	user choice
Living Life to the full In	10.1016/S2215-0366(16)00083-3	1	2016	Blended	12	RCT	307	Module-based	No	0		0		0		0	
Living to the full	10.1192/bjp.bp.114.146068, 10.1016/j.brat. 2015.06.014	2	2016	Guided	9-12	RCT	321	Module-based	No	0		0		0		1	user choice, rule-based
makora AG	10.3389/fdgth.2020.00004	1	2020	Unguided	12	RCT (with vs.	168	Module-based	No	0		0		0		1	rule-based
Marigold	10.1016/j.jad.2019.07.049, 10.2196/25922	2	2021	Guided/Unguide d (Trial Arms)	5	RCT, Prepost	644	Other	No	0		0		0		1	rule-based
MBM	10.1007/s10608-019-10042-x	1	2020	Unguided	0,5	RCT (no control)	153	Memory Bias Modification	No	0		0		0		0	
MCT & More	10.2196/26498	1	2021	Unguided	4	RCT	200	Single Exercises	No	1	user choice	0		0		0	

Meta Data										Content Variability		Order Variablity		Guidance Variability		Comunication Variability	
Name	Papers	PaperCount	Year (newest)	Type	Duration in weeks	Study Type	Sample Size	Content	Direct Comparison	Content variable	Source of Variability	Order variable	Source of Variability	Type variable	Source of Variability	Comunication variable	Source of Variability
mentalis phoenix	10.2196/16643, 10.1186/s40814-021-00799-y	2	2021	Unguided/Blended	2	RCT	48	Other	No	0		0		0		0	
Meru Health	10.1016/j.jad.2021.02.007 10.1007/s10484-020-09458-z 10.2196/25808 10.1016/j.invent.2021.100408	4	2021	Guided	8	RCT/Pre post	349	Module-based	No	0		0		0		0	
Mobile Sensing and S	10.2196/mhealth.5960	1	2016	Unguided	2	Feasibility	126	Other	No	1	ML model	0		0		0	
MOOD	10.2196/14240	1	2019	Unguided	6	RCT	64	Module-based	No	0		1	user choice	0		1	rule-based
MoodBuster	10.1159/000503408	1	2019	Blended	13	Case Report	1	Module-based (lessons)	No	1	user choice, provider choice	1	user choice, provider choice	1	rule-based, user choice - provider choice	0	
Moodgym	10.1016/j.ajp.2017.11.007, 10.3310/hta20890, 10.3389/fpsyt.2017.00032, 10.1177/2043808719842502,10.1136/bmj.h5627, 10.1016/j.jad.2018.06.008	6	2019	Guided/Unguided	4 - 12 - 6	RCT	1524	Module-based	No	0		0		1	user choice, provider choice	0	
MoodHacker	10.2196/mhealth.4231	1	2016	Unguided	6	RCT	150	Other	No	0		0		0		0	
MoodTracker	10.1016/j.jad.2021.05.021	1	2021	Unguided	3	RCT	58	Mood Monitoring	No	0		0		0		0	
MORIBUS	10.1145/3329189.3329214	1	2019	blended	4	Feasibility	7	Other	No	0		0		0		0	
MP	10.2196/15845	1	2020	Unguided	4-8	RCT	54	module-based	No	0		0		0		1	rule-based
MUBS	10.1145/3313831.3376879	1	2020	Unguided	8	Feasibility	17	RecommenderSys	No	1	user choice, ML model	0		0		0	
My Food & Mood	10.2196/24871	1	2021	Unguided	8	Cohort Study	614	Other	No	0		0		0		0	
myStrength	10.2196/resprot.7203	1	2017	Unguided	26	RCT	78	Other	No	1	patient choice	0		0		0	
PAPP (before: iTAU)	10.2196/15845	1	2020	Blended	4-8	RCT	56	Module-based	No	0		0		0		1	rule-based
Plattform	10.1145/3313831.3376510	1	2020	blended	Unclear	Cohort Study	17	Other	No	1	provider choice	0		0		0	
PRIME-D	10.1002/da.22624	1	2017	Guided	8	Cohort Study	36	Other	No	0		0		1	provider choice, user choice	0	
Push-D	10.3390/bs8040036	1	2018	Guided	10	Cohort Study	78	Module-based	No	1	user choice, rule based	0		0		1	rule based
Resilience Training	10.1136/ebmental-2022-300455	1	2018	Unguided	8	RCT (no control)	1093	Other	No	0		0		0		1	rule-based
Self-administered Psy	10.1177/20552076221091353	1	2022	Unguided	Unclear	Feasibility (prepost)	110	Unclear	No	1	rule-based, user choice	0		0		1	rule-based
Smiling is Fun	10.2196/jmir.5695, 10.2147/NDT.S130994	2	2017	Guided/Unguided	10	RCT	274	Module-based.	No	0		0		0		1	rule-based
Space from Depressio	10.1016/j.brat.2015.10.005, 10.1016/j.invent.2016.06.007, 10.2196/13392, 10.1016/j.invent.2018.11.005	4	2020	Guided	8	RCT/UX	210	Module-based	No	0		1	user choice	0		1	rule-based
SPSRS	10.1016/j.jad.2020.12.104, 10.1111/eip.12540	2	2021	Unguided	5	RCT	38	Youtube	No	0		0		0		1	rule-based
Step-By-Step	10.1371/journal.pmed.1004025, 10.1136/ebmental-2021-300416, 10.1016/j.invent.2021.100380	4	2022	Guided	5-8	RCT	681	Module-based	No	1		0		1	rule-based	1	user choice
Super@	10.2196/27410	1	2021	Guided	14	Cost Effectiveness Study - RealWorldData	229	Module-based	No	1	user choice	0		1	rule-based	0	
SuperBetter	10.1089/g4h.2014.0046	1	2015	Unguided	4	RCT	94	Other	No	1	rule-based	0		0		0	
Taking Control	10.2196/jmir.4861	1	2016	Guided	8	RCT	136	Module-based	No	0		0		1	rule-based	0	
The Journal	10.2196/mental.8510	1	2018	Blended	12	RCT	35	Module/Lesson based	No	0		0		1	rule-based	0	
The Mood Course	10.1016/j.janxdis.2015.08.002	1	2015	Guided/Unguided	8	RCT	141	Module-based	No	0		0		1	provider choice	1	rule-based
The Sadness Program	10.1002/da.22590, 10.1016/j.invent.2017.05.001, 10.1002/acr.23257, 10.1016/j.jad.2015.02.026, 10.1016/j.jad.2014.08.038	5	2022	Guided	6	RCT/Prepost Routine	681	Module-based	No	0		0		1	rule-based	0	
ThinkFeelDo	10.1016/j.brat.2019.103485, 10.4108/icst.pervasivehealth.2015.260115	2	2019	Guided	8/20	RCT, PrePost	145	Lessons-based	No	0		0		1	provider choice, user choice, rule-based	1	user choice
Thought Challenger	10.1037/ser0000261, 10.2196/humanfactors.7951	2	2019	Guided	6	RCT, Usability Study	30	Cognitive Restructuring Exercise	No	0		0		0		1	rule-based, user choice
Thrive	10.2196/14754, 10.7812/TPP/21.183	2	2022	Unguided	8	RCT	329	Module-based.	No	1	user choice, rule-based	0		0		0	
Thrive (Singapore)	10.3389/fpsyg.2021.668384	1	2021	Blended	4	RCT	29	Module-based	No	0		0		0		0	
Unnamed_A	10.2196/jmir.9890	1	2018	Blended	10	Other	45	Module-based	No	1	provider choice	0		0		0	
Unnamed_B	10.1186/s12888-017-1248-8	1	2017	Unguided	5	RCT	326	Single Exercise	No	0		0		0		0	
Unnamed_C	10.1186/s12888-017-1248-8	1	2017	Unguided	5	RCT	323	Single Exercise	No	0		0		0		0	
Unnamed_D	10.1111/bjc.12287	1	2021	Blended	2	RCT	25	Other	No	0		0		0		0	
Unnamed_E	10.1177/2150132720971158	1	2020	Blended	8	RCT	11	Other	No	0		0		0		0	
Unnamed_F	10.1016/j.jad.2020.07.087	1	2020	Unguided	2	RCT (program versions - Pre-Post)	206	Other	No	0		0		0		0	
Unnamed_G	10.1016/j.invent.2018.06.004	1	2018	Unguided	4	RCT	65	Single Module.	No	0		0		0		0	
Unnamed_H	10.1371/journal.pone.0126559	1	2015	Blended	9	RCT	46	Other	No	0		0		0		0	

Meta Data										Content Variability		Order Variablity		Guidance Variability		Comunication Variability	
Name	Papers	PaperCount	Year (newest)	Type	Duration in weeks	Study Type	Sample Size	Content	Direct Comparison	Content variable	Source of Variability	Order variable	Source of Variability	Type variable	Source of Variability	Comunication variable	Source of Variability
Unnamed_I	10.2196/JMIR.4861	1	2016	Guided	5	RCT	136	Single Module ('session)	No	0		0		1	rule-based	0	
Unnamed_J	10.1016/j.jad.2017.03.018	1	2017	Guided	12	RCT	121	Behvarioual Act.	No	0		0		0		0	
Unnamed_K	10.1016/j.jad.2017.03.018	1	2017	Guided	12	RCT	112	Other	No	0		0		0		0	
Unnamed_L	10.1016/j.invent.2019.100247,10.1016/j.invent. 2019.100274, 10.1016/j.invent.2016.11.002, 10.1002/jclp.22753, 10.1007/s10943-017-0503-0	2	2019	Guided	8	RCT	233	Module-based.	No	0		0		1	rule-based	0	
Unnamed_M	10.1192/bjp.bp.114.160101	1	2015	Guided	12	RCT	317	Module-based	No	1	rule based, user choice, provider choice	0		1	rule-based, user choice	0	
Unnamed_N	10.2196/15312	1	2020	Unguided	2	RCT	32	Mindfulness	No	1	user choice	0		0		0	
Unnamed_O	10.2196/15312	1	2020	Unguided	2	RCT	37	iBA	no	1	user choice	0		0		0	
Unnamed_P	10.2196/10698	1	2018	Guided	8	RCT	14	Exercise	No	1	provider choice, rule based	0		0		0	
UTSMed	10.1016/j.jad.2016.05.045	1	2016	Unguided	Unclear	RCT	618	website	No	0		0		0		0	
WPAPP	10.9741/23736658.1044	1	2016	Unguided	12	RCT	18	Other	No	1	rule based	0		0		0	
