# Supplementary material for: Personalization strategies in digital mental health interventions: a systematic review and conceptual framework for depressive symptoms
Source: Front Digit Health. 2023 May 22;5:1170002. doi: 10.3389/fdgth.2023.1170002 (PMC10239832; doi:10.3389/fdgth.2023.1170002)
Supplement: Supplementary file 3 [file Datasheet3.pdf]

Appendix 3: Search Strings

Search terms and the search strategy were evaluated and iterated at an early step before the finalization of the PROSPERO registration. Specifically, we evaluated the tradeoff of sensitivity and specificity in form of number and quality of results for different search strategies.

SCOPUS:
(TITLE (depress*)) AND (TITLE (smartphone) OR TITLE (mobile) OR TITLE (phone) OR TITLE (app) OR TITLE (web) OR TITLE (internet) OR TITLE (digital) OR TITLE (online) OR TITLE (web-based) OR TITLE (icbt) OR TITLE (computer)) AND PUBYEAR > 2014

Pubmed:
((("Depression"[Mesh] OR "Depressive Disorder"[Mesh]) OR (depress*[ti])) AND ((smartphone*[ti]) OR (mobile[ti]) OR (phone[ti]) OR (app[ti]) OR (web[ti]) OR (internet[ti]) OR (digital[ti]) OR (online[ti]) OR (web-based[ti]) OR (iCBT[ti]) OR (computer[ti]) OR ("digital technology"[Mesh]) OR ("mobile applications"[Mesh]) OR ("Smartphone"[Mesh]))) AND (("2015"[Date - Publication] : "3000"[Date - Publication]))

Psycinfo:
(MA depress* OR TI depress*) AND (TI (smartphone OR mobile OR phone OR app OR web OR internet OR digital OR online OR web-based OR icbt OR computer) OR MA (Smartphone OR mobile application OR digital technology))
